# Seroprevalence study of brucellosis in wild boar hunted for private consumption in northeast Portugal

**DOI:** 10.1007/s11259-024-10317-z

**Published:** 2024-02-05

**Authors:** Zita Martins Ruano, Teresa Letra Mateus, Ana Chorense, Sérgio Santos-Silva, Madalena Vieira-Pinto

**Affiliations:** 1 Veterinary and Animal Research Center (CECAV), University of Trás-os-Montes e Alto Douro, Quinta de Prados, Apartado 1013, Vila Real, 5001-801 Portugal; 2 Associate Laboratory for Animal and Veterinary Sciences (AL4AnimalS), Lisbon, Portugal; 3 Center for Research and Development in Agrifood Systems ad Sustainability (CISAS), Escola Superior Agrária, Instituto Politécnico de Viana do Castelo, Viana do Castelo, 4900-347 Portugal; 4 EpiUnit – Instituto de Saúde Pública da Universidade do Porto, Laboratório para a investigação integrativa e translacional em saúde populacional (ITR), Universidade do Porto, Rua das Taipas, nº135, 4050-091 Porto, Portugal; 5 Department of Veterinary Sciences, University of Trás-os-Montes e Alto Douro, Quinta de Prados, Apartado 1013, 5001-801 Vila Real, Portugal; 6 School of Medicine and Biomedical Sciences (ICBAS), University of Porto, Porto, Portugal

**Keywords:** *Brucella* spp., One health, *Sus scrofa*, Zoonosis

## Abstract

Brucellosis is an important infectious disease caused by bacteria of the genus *Brucella*. In the northeast region of Portugal, infection with *Brucella melitensis* is endemic in small ruminants, and there are also humans’ cases. However, the epidemiological role of the wild boar in the dynamics of this disease in this region is unknown. In this study, a total of 332 blood samples were collected from wild boar hunted in thirty-six hunting areas during the 2022/2023 hunting season. All were taken by the hunters for private consumption, with no evisceration or examination in the field. Serum samples were tested by indirect ELISA (i-ELISA). It was observed that 88 wild boars were exposed to *Brucella* spp., pointing to a seroprevalence of 26.5% (95% CI: 21.8 – 31.3%). This high prevalence underlines the importance that wild boar may have in the dynamics of this disease in the region and its potential transmission to other animals, and to humans (for example, during the handling of carcasses). Increased awareness and knowledge of brucellosis in wild boar is essential for the implementation of effective practices and habits and, consequently, for the control and prevention of this important zoonosis.

## Introduction

*Brucella* species are one of the main pathogenic zoonotic agents that infect domestic animals, including dogs and wild animals (Godfroid et al. 2013), being the cause of serious public health and economic threats (Godfroid 2017). In 2020, 128 confirmed brucellosis cases in humans were reported in the European Union. In Portugal nine cases were confirmed. According to the European Food Safety Authority (EFSA), eleven member states reported information on the *Brucella* species for human cases with *B. melitensis* being the species most reported, followed by *B. abortus* and *B. suis* (EFSA 2021). In Portugal, brucellosis is one of the three most frequent zoonosis, but studies on prevalence are scarce and focused only on small ruminants (Coelho et al. 2007, 2019; Castelo and Simões 2019). Human cases are reported in all regions of continental Portugal, as shown in the 2014–2018 report of the General Directorate of Health (DGS). These human cases are mainly associated with *B. melitensis* (Ferreira et al. 2012a; Pelerito et al. 2017; DGS 2018).

Brucellosis still has a high incidence in some regions of Portugal, especially in northeast Portugal, where brucellosis is endemic in sheep and goats (Castelo and Simões 2019; Coelho et al. 2019). Portugal applies specific regulations and measures to eradicate the disease, however, brucellosis has continued to be an endemic disease where *B. melitensis* biovars 1 and 3 and *B. abortus* biovars 1 and 3 are the prevailing animals’ species (Ferreira et al. 2013). According to the eradication and control plan for the disease in Portugal, at the 31st December 2017, 82.7% of the 52 herds who had been infected were from the north of the country (DGAV 2019).

Vaginal excretions and aborted material from infected animals are the major sources of contamination in feeding areas (pastures and water), constituting the main sources of infection among animals (Coelho et al. 2019). The disease is associated with reproductive losses in animals worldwide (Godfroid 2017), but in general, most infected animals do not demonstrate clinical illness on visual examination (Olsen and Tatum 2016). Humans are foremost infected through consumption of unpasteurized dairy products (Whatmore 2009) or through direct contact with infected animals, their excretions and/or carcasses (Mailles et al. 2017).

Brucellosis is known to be an important disease in wildlife and all *Brucella* species can also infect wild species (Meng and Lindsay 2009; Godfroid et al. 2010). Furthermore, it is known that having a reservoir of the disease in wildlife can complicate eradication efforts (Whatmore 2009). Within wildlife, large game species, like wild boar (*Sus scrofa*) which may be in closer contact with humans, may constitute an important threat in the transmission of zoonotic diseases, that must be addressed. Wild boar is known as an important reservoir of *B. suis* (Ruiz-Fons et al. 2006; Closa-Sebastià et al. 2010; Muñoz et al. 2010; Wu et al. 2011; Grégoire et al. 2012; Szulowski et al. 2015; Pilo et al. 2015; Mailles et al. 2017; Pyskun et al. 2019; Kamga et al. 2020; Montagnaro et al. 2020; van Tulden et al. 2020; Lambert et al. 2021). While it is still unknown, we hypothesized that there is a high incidence of *Brucella* in wild boar from Portugal. It is estimated that in Portugal there is a population of 277,385 wild boars. Through the Wild Boar Strategic and Action Plan in Portugal, can be seen that in Portugal wild boar populations are increasing, both in number and distribution (Instituto Nacional da Conservação da Natureza e Florestas (ICNF) 2022). In the northeast region of Portugal, wild boar is currently the most important wild species hunted. Furthermore, there is evidence of wild boar/domestic animal (e.g., pigs raised outdoor, hunting dogs)/humans sympatric interactions that may contribute to the interspecies transmission of this agent. Under this epidemiological scenario, what could be the role of wild boar? This study aimed to identify the seroprevalence and spatial distribution of brucellosis in wild boar hunted in the northeast of Portugal increasing knowledge about this disease in wild boar in Portugal.

## Materials and methods

### Ethical approval

Our study did not require ethical approval, because all samples were collected from wild boar legally hunted. No live animals were used for this study.

### Area of study

**Fig. 1:**
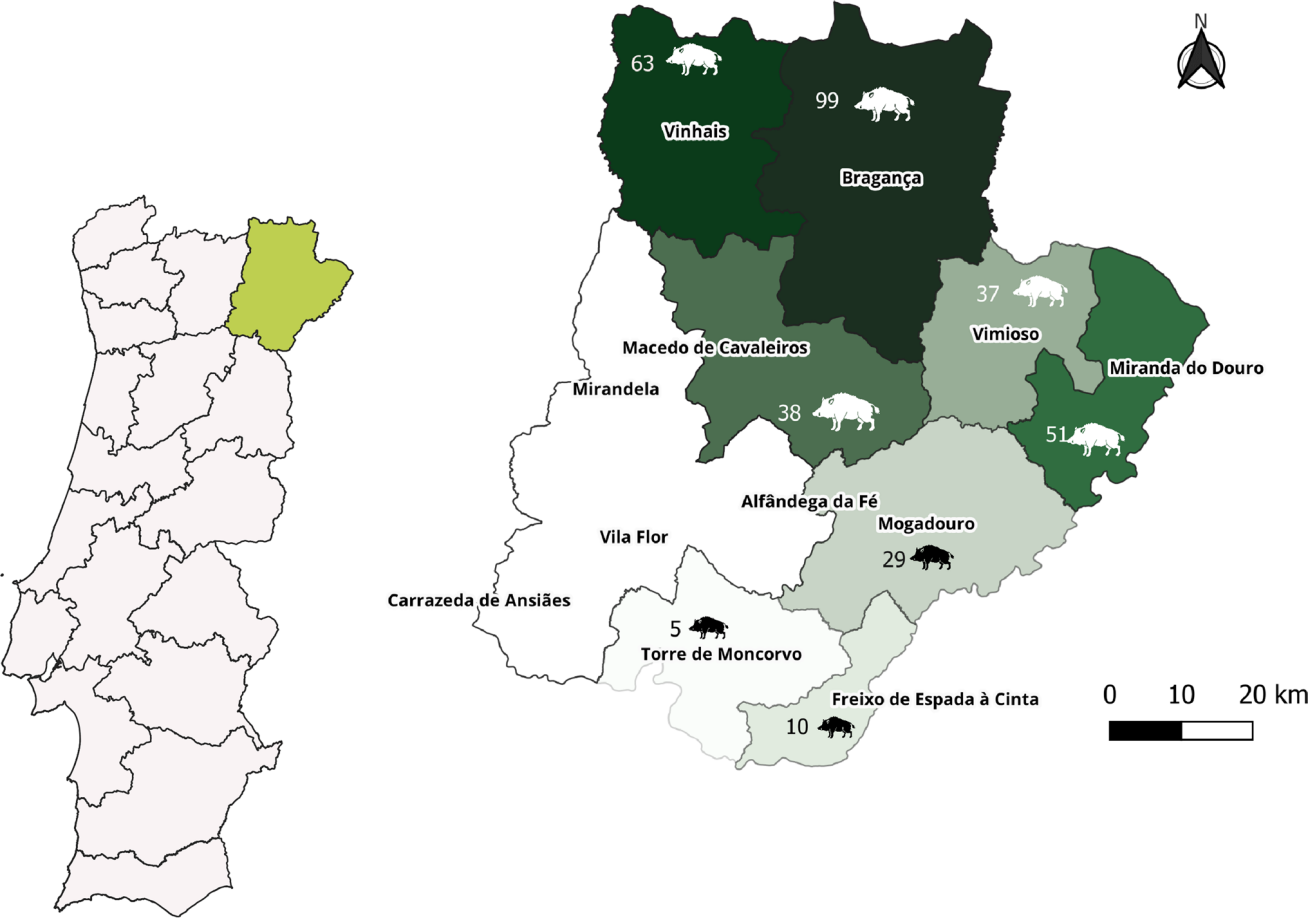
The map of Portugal showing the area of study (Bragança district) and the number of wild boars sampled

The present study was performed in the district of Bragança (Fig. 1), in the northeast of Portugal, where brucellosis is an endemic disease (DGAV 2019).

The territory is very mountainous with abundant wild species, especially wild boar. Oak acorns represents the basic diet of wild boar (Sütő et al. 2020) and the study area is favorable for wild boar maintenance. The region is characterized mainly by oaks, chestnut trees, shrub vegetation like heather *Erica* spp., gum rockrose and fragmented by cultivated fields.

There are several herds of ruminants and domestic pigs raised outdoor favoring possible contact between wildlife directly or through the common natural resources (food and water).

### Sampling and laboratory analysis

A cross-sectional study was carried out during the 2022/2023 hunting season (October – February) to determine the seroprevalence of *Brucella* spp. in wild boar (*Sus scrofa*). Thirty-six hunting associations from the area of study were contacted to collaborate in the study. All hunting associations accepted to participate in the study.

A non-probabilistic sampling method (convenience sampling) was used in this study. A total of 332 blood samples from wild boar were collected in eight municipalities (Bragança, Freixo de Espada à Cinta, Macedo de Cavaleiros, Miranda do Douro, Mogadouro, Torre de Moncorvo, Vimioso and Vinhais) of the Bragança district. Blood samples were obtained using a 10 mL syringe, tubes containing clot activator (BD Vacutainer®, Plymouth, UK) and a 80 mm long needle (1 × 280 mm, BOVIVET, Kruuse®, Denmark), by ocular puncture, described by Arenas-Montes et al. (2013) (Arenas-Montes et al. 2013). Samples were refrigerated to be taken to the laboratory.

No animals were eviscerated and examined after the hunt. All were taken by the hunters for private consumption to different parts of the country.

After coagulation the blood samples were centrifugated and serum stored at -20ºC until analyses. The samples were analysed for antibodies against *Brucella* spp. using a multi-species i-ELISA test kit (ID Screen® Brucellosis Serum Indirect Multi-species, ID vet Innovate Diagnostics, Grabels, France), following the instructions of the manufacturer. The optical density (OD) was read at 450 nm and results were evaluated by calculating the S/P [OD_sample_ - OD_NC_] / [OD_PC_ - OD_NC_] x 100. Samples with S/P%≤110% were considered negative, 110–120% doubtful, and ≥ 120% positive.

### Statistical data analysis

Seroprevalence of *Brucella* spp. was estimated from the ratio of positive samples to the total number of samples analysed. The 95% confidence interval (CI) for seroprevalence proportions was calculated.

## Results

Seven out of eight municipalities were positive for *Brucella* spp. antibodies. Eighty-eight wild boars were exposed to these bacteria, pointing to a seroprevalence of 26.5% (95% CI: 21.8 – 31.3%). The number of positive and doubtful results and seroprevalence of *Brucella* spp. in wild boar per municipality is shown in Table 1.

**Table 1:** Seroprevalence of brucellosis in wild boar from 8 municipalities of Bragança district

Municipality	Number of tested animals	Number of doubtful results	Number of positives results	Seroprevalence (%)	95% CI (%)
Bragança	99	0	22	22.2	14.0–30.4
Freixo de Espada à Cinta	10	0	5	50.0	19.0–81.0
Macedo de Cavaleiros	38	1	13	34.2	19.1–49.3
Miranda do Douro	51	0	7	13.7	4.3–23.2
Mogadouro	29	0	7	24.1	8.6–39.7
Torre de Moncorvo	5	0	0	0.0	0.0
Vimioso	37	1	10	27.0	12.7–41.3
Vinhais	63	0	24	38.1	26.1–50.1
Total	**332**	**2**	**88**	**26.5**	**21.8–31.3**

The seroprevalence of antibodies to *Brucella* spp. was 50.0% (95% CI: 19.0 – 81.0%) in wild boar from Freixo de Espada à Cinta municipality, followed by Vinhais (38.1%, 95% CI: 26.1 − 50.1%), Macedo de Cavaleiros (34.2%, 95% CI: 19.1 − 49.3%), Vimioso (27.0%, 95% CI: 12.7 − 41.3%), Mogadouro (24.1%, 95% CI: 8.6 − 39.7%), Bragança (22.2%, 95% CI: 14.0 − 30.4%) and Miranda do Douro (13.7%, 95% CI: 4.3 − 23.2%). We had two results doubtful in Macedo de Cavaleiros and Vimioso municipalities and no positive samples were registered in the municipality of Torre de Moncorvo.

## Discussion

Brucellosis in wildlife has been neglected (Lambert et al. 2021). According to Ferreira et al. (2017) swine brucellosis is an emerging disease in Europe which has been associated with the existence of extensive swine farms and the increase number of infected wild boar. Ferreira et al. (2012b) tested tissues samples from 918 hunter-harvested wild boars across Portugal, and 63 animals (6.9%) were found to be infected with *B. suis* biovar 2. The maintenance and spread of *B. suis* biovar 2 in Europe are a dynamic process, which depends on the natural expansion of the wild boar as the principal wild reservoir of infection, playing a critical role for the transmission of infection to pigs (Muñoz et al. 2019). Data on brucellosis in wild boar in Portugal are scare, for this reason and in a region where brucellosis is endemic in small ruminants, and there are also human cases, this study aimed to determine the seroprevalence of brucellosis in wild boar in the district of Bragança, Portugal, during the 2022/2023 hunting season, using a multi-species i-ELISA test kit. From a total of 332 serum samples, 88 (26.5%, 95% CI: 21.8 – 31.3%) were positive to *Brucella* spp. antibodies and two doubtful results were also obtained.

In contrast to what happens in Portugal, there are some studies on the seroprevalence of brucellosis in wild boar in Spain, neighboring country of Portugal. In 2006, Ruiz-Fons et al. (2006), studied the seroprevalence of six reproductive disease pathogens in wild boar females and revealed a seroprevalence of 29.7% for *Brucella* spp. In other study, wild boar showed a high prevalence of brucellosis (33.0%) in all Spanish territory (Muñoz et al. 2010). A lower seroprevalence was found in northeast of Spain; this study was conducted by Closa-Sebastià et al. (2010) in 2010 and detected in 28 of the 256 (10.9%) wild boar *Brucella* antibodies. In the rest of Europe, some studies have also been carried out to clarify the situation of brucellosis in wild boar. High seroprevalences were detected in Belgium and Switzerland, with 54.9% and 28.8%, respectively (Wu et al. 2011; Grégoire et al. 2012). In Italy, antibodies to *Brucella* spp. were found in wild boar in Sardinia region (2015) and in Campania region (2020) with seroprevalences of 6.1% and 13.6%, respectively (Pilo et al. 2015; Montagnaro et al. 2020). In Netherlands, the prevalence ranged from 4.1–11.6%, in different provinces (van Tulden et al. 2020). Poland and Ukraine also had lower seroprevalences, registering 24.5% and 11.3%, respectively (Szulowski et al. 2015; Pyskun et al. 2019).

This study revealed that in the northeast of Portugal the role of wild boar could be of relevance, possibly serving as reservoir of brucellosis and spillover infections to sympatric domestic animals and humans.

Brucellosis in wild boar can be widespread in the northeast of Portugal, thus representing an important threat for domestic pigs, in particular, Bísaro pig, an autochthonous breed in the northeast of Portugal. This breed is mostly reared in a semi-extensive system where breeders have pigsties but the animals are still free to spend most of their time roaming the adjacent parks. Therefore, there is a higher risk of interactions between pigs outdoors and wild boar in the study area. In Switzerland, swine brucellosis was detected on two outdoor pig farms after contact with wild boar (Wu et al. 2012). In Portugal, Ferreira et al. (2017), published a report on the genetic diversity of *B. suis* isolates showing the importance of considering spillover of *B. suis* biovar 2 infection from wild boar to pigs, sheep, and cattle.

A study carried out in France demonstrates, for the first time, *B. suis* biovar 2 infection in dogs, where contact with wildlife was possible (Girault et al. 2023). Dogs are infected when in contact with body fluids and tissues from infected wild boar. Brucellosis should be considered in the differential diagnosis of abortion, testicular/epididymal enlargement, lameness and discospondylitis (James et al. 2017). The prevalence found in this study should raise awareness among owners of hunting dogs about possible exposure to the disease.

The seroprevalence found highlights that brucellosis in wild boar may represent a significant threat to public health, as it was previously referred for other geographical regions. In France, seven cases of *B. suis* in humans have been reported and all patients had direct contact with wild boar while hunting or preparing wild boar meat for consumption, which proves the occupational threat to humans, principally hunters (Mailles et al. 2017). Uninspected or unexamined game meat for private consumption can pose a health risk, and despite the risk, there is no mandatory initial examination of the carcasses of wild boar hunted in northeast of Portugal. All 332 carcasses were not eviscerated and examined after the hunt. All were taken by the hunters for private consumption (with no inspection). These results suggest that hunters or other people during the carcasses’ handling may be exposed at home if no protective measures are adopted. The low brucellosis awareness and knowledge level and incorrect practices in handling, cooking and preserving animal-based food, poses a great threat to public food safety (Mailles et al. 2017).

Also, due to the fact that hunters take wild boar for private consumption, the disposal of by-products may not be done correctly. A study carried out in Portugal during the three hunting seasons (from the year 2020 to 2023) by Abrantes et al. (2023), concluded that 11% of hunters or managers of hunting areas do not correctly dispose of by-products, endangering public health. Proper disposal of by-products is critical to preventing the spread of brucellosis and other diseases (Sannö et al. 2018).

Knowledge of the epidemiology of brucellosis is of paramount importance for the protection of public health, particularly among high-risk groups such as hunters. Knowledge/training allows people to take protective measures and to actively participate in disease control programs by actively contributing to the development of brucellosis control strategies (Zhang et al. 2019).

Our study had several limitations due to the lack of initial examination of wild boar carcasses in this region. For this reason, there was an unequal proportion of animals’ samples in each region, which influences the results obtained. For example, the results in the Torre de Moncorvo region are probably due to the low number of samples analysed and not to the absence of *Brucella* circulation. However, in studies of wildlife we can not predict sampling more accurately. The lack of data on risk factors also generated insufficient data to provide the basis for a representative statistical description and analyses.

The role of wild boar can be of great importance, but is often largely neglected. Furthermore, wildlife brucellosis inspection is not mandatory and data are scarce. This study aimed to get insights and increase knowledge into the occurrence of brucellosis in wild boar hunted in a Portuguese region where brucellosis is endemic in livestock.

This information should trigger increased attention from the competent national veterinary authorities who should encourage surveillance and control actions for this important zoonotic disease. Furthermore, more information/training should be given to hunters so that they can implement effective protection measures. Protection should be used while handling wild boar animals and awareness of by-products elimination should be raised among hunters.

In future, it would be highly desirable to collect more accurate epidemiological information on the prevalence of wild boar brucellosis and its etiology.

In a screening situation, one important issue is the interpretation of doubtful results obtained with the i-ELISA. With these results and in a future analysis of the samples, doubtful results will be screened again, to be eliminated.

## Conclusions

Our study is the first report on the seroprevalence of *Brucella* in wild boar hunted in Bragança in Portugal.

Given the fact that in wild boar hunts in Portugal the initial examination of the hunted animal is not always performed (Abrantes et al. 2023), the risk of transmission of brucellosis to humans, hunting dogs and livestock can be considered high. Attention should be given to biosecurity measures for wild boar hunters and livestock farms to prevent brucellosis infection. Also, health education about the disease for high-risk groups, like hunters, could be of capital importance. Furthermore, more information/training should be given to hunters so that they can implement effective protection measures.

*B. suis* biovar 2 infection spillover from the wild boar to cattle, small ruminants, and dogs need to be assessed, as this will interfere in the epidemiology.

This information should trigger increased attention from the Competent National Veterinary Authority (DGAV) who should encourage surveillance and control actions for this important zoonotic disease.

More studies on the identification of brucellosis are essential to provide epidemiological data for control of this zoonosis in the northeast of Portugal.
